# Case Report: A successfully managed case of SMARCA4-deficient undifferentiated gastric carcinoma

**DOI:** 10.3389/fonc.2026.1775517

**Published:** 2026-02-24

**Authors:** Rui Peng, Yun Shi, Pingping Wu, Jia Chen, Gang Li

**Affiliations:** 1 Department of General Surgery, The Affiliated Cancer Hospital of Nanjing Medical University & Jiangsu Cancer Hospital & Jiangsu Institute of Cancer Research, Nanjing, Jiangsu, China; 2 Department of Oncology, The Affiliated Cancer Hospital of Nanjing Medical University & Jiangsu Cancer Hospital & Jiangsu Institute of Cancer Research, Nanjing, Jiangsu, China

**Keywords:** BRG1, immunotherapy, SMARCA4, targeted therapy, undifferentiated gastric carcinoma

## Abstract

We report a case of SMARCA4-deficient poorly-to-undifferentiated gastric carcinoma. The patient underwent surgical resection. Postoperative treatment combined chemotherapy, immunotherapy, and targeted therapy. The regimen included ifosfamide, liposomal doxorubicin hydrochloride, cadonilimab, and anlotinib capsules. Follow-up to date shows no tumor recurrence or metastasis. The postoperative relapse-free survival (RFS) has now reached 33 months. Primary gastric SMARCA4-deficient undifferentiated tumors carry a poor prognosis, with survival typically under one year. Here we present a successfully treated case of this tumor type originating in the stomach. This case may serve as a reference for future management of such tumors.

## Introduction

The protein BRG1, encoded by SMARCA4, serves as the ATPase catalytic subunit of the SWI/SNF complex (1). It is involved in key biological processes including cell differentiation, proliferation, and DNA repair (1). Loss of its function disrupts chromatin remodeling and causes transcriptional dysregulation and genomic instability (2). This deficiency is strongly associated with various malignancies, such as lung and ovarian cancers (3, 4). SMARCA4-deficient tumors often exhibit poorly differentiated morphology. Primary gastric cases are extremely rare and correlate with a poor prognosis (5, 6). Currently, no standard treatment exists. We report a case of SMARCA4-deficient gastric carcinoma and review its management and clinical outcome.

## Case presentation

**Figure 1:**
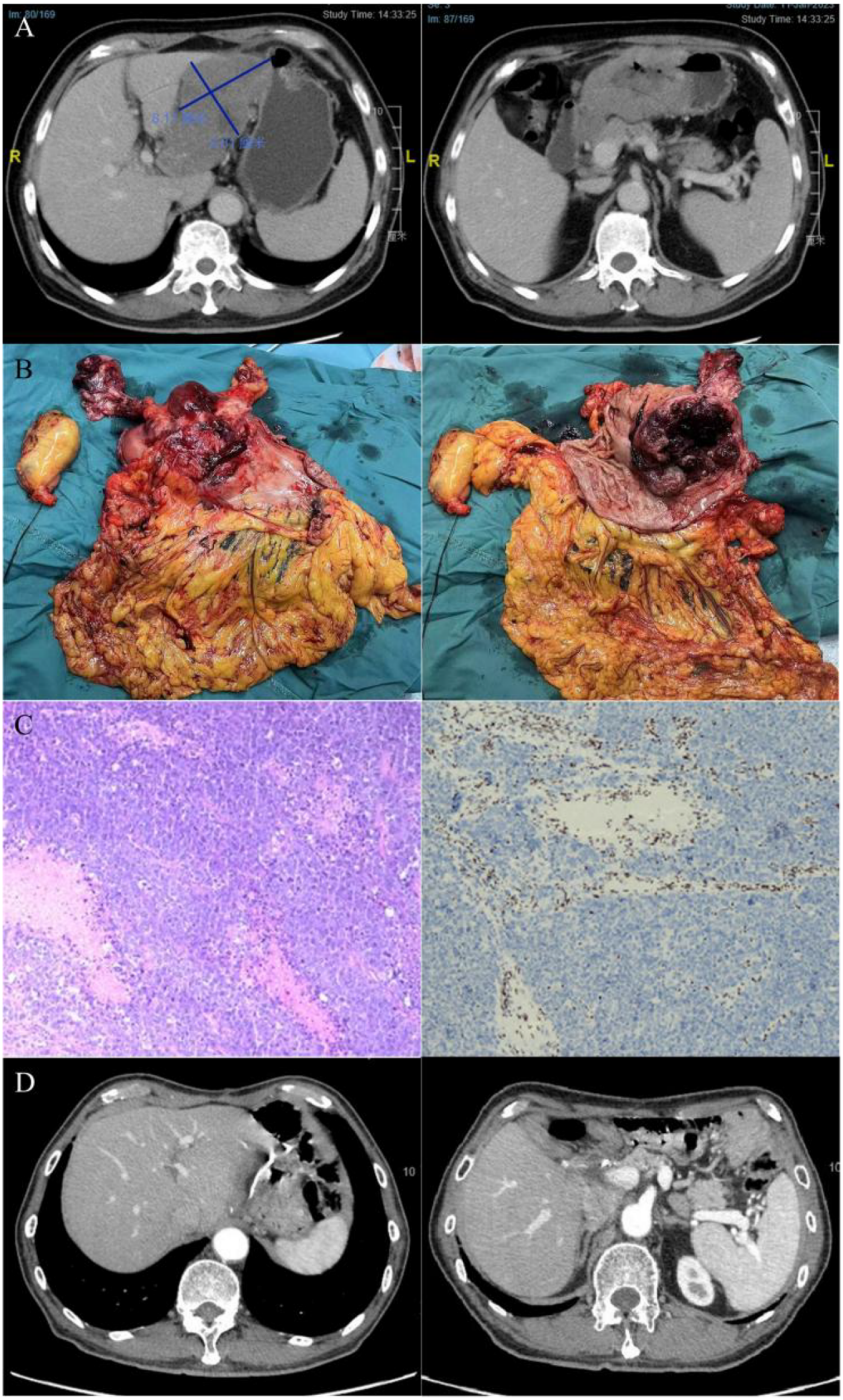
**(A)** Contrast-enhanced CT scan (Jan 11, 2023) after two cycles of neoadjuvant therapy, indicating disease progression. The image reveals marked thickening of the gastric antral wall with an irregular serosal surface, alongside multiple enlarged and partially confluent lymph nodes within the hepatogastric ligament. No significant lymphadenopathy was identified in the peritoneal cavity or retroperitoneum. **(B)** Macroscopic view of the resected specimen. **(C)** Representative histological sections showing H&E staining (left) and concurrent SMARCA4 (BRG1) immunohistochemistry (right), the latter confirming loss of expression in tumor cells. **(D)** Follow-up imaging after upper abdominal surgery (subtotal gastrectomy), with no signs of tumor recurrence or metastasis.

A 68-year-old male patient presented to the hospital in November 2022 with a two-week history of fatigue and shortness of breath following physical activity, and melena. Computed Tomography (CT) revealed a mass in the gastric antrum with enlarged lymph nodes in the hepatogastric space. The largest cross-sectional dimension of the antral mass measures approximately 7.89 cm × 6.72 cm. Biopsy pathology confirmed a malignant tumor at the lesser curvature of the gastric antrum (suspected poorly differentiated neuroendocrine carcinoma). The clinical stage was determined as cT4aN3M0. The patient received two cycles of neoadjuvant therapy with etoposide, lobaplatin, and atezolizumab. Follow-up CT showed the largest cross-sectional dimension of the mass measured approximately 8.17 cm × 6.81 cm, indicating tumor progression (**Figure 1A**). On January 17, 2023, the patient underwent radical distal gastrectomy (**Figure 1B**). Postoperative histopathological examination confirmed gastric malignancy. IHC demonstrated partial positivity for vimentin (Vim), whereas BRG1 was negative (**Figure 1C**). IHC demonstrated positivity for PMS2, MSH2, MSH6, MLH1, Syn, CD56 (partial positivity), INSM-1 (focal positivity), and INI1, with partial Vim expression. PD-L1 showed a CPS of 2, whereas PD-1, HER2, Claudin18.2, E-cadherin, SALL4, AFP, GPC-3, CD10, LCA, CK7, CK20, Villin, AE1/AE3, S100, CK5/6, P40, P63, HepPar-1, TTF1, and BRG1 were negative. The Ki-67 proliferation index reached 70%, with mutant p53 pattern and TP positivity in 20% of cells. Genetic testing of the postoperative specimen revealed a SMARCA4 mutation. The patient was diagnosed with a SMARCA4-deficient, poorly-to-undifferentiated gastric tumor. No standard regimen is currently recommended in guidelines. Following a multidisciplinary team (MDT) discussion, a combined regimen was proposed: chemotherapy (ifosfamide + liposomal doxorubicin), immunotherapy (cadonilimab), and targeted therapy (anlotinib). Cadonilimab is a PD-1/CTLA-4 bispecific antibody (7). Anlotinib is a multi-target tyrosine kinase inhibitor (8). The patient received two cycles of combination therapy starting February 16, 2023 and March 9, 2023. The regimen comprised: Ifosfamide 2g (days 1, 2, 8, 9); Liposomal doxorubicin hydrochloride 25mg (days 1, 8); Cadonilimab 250mg (days 1, 8); Anlotinib capsules 8mg (daily from day 1 to 14). Treatment cycles were repeated every 3 weeks.For cycles 3 (April 3, 2023) and 4 (May 5, 2023), the anlotinib dose was increased to 12mg. Chemotherapy was subsequently discontinued. The patient underwent two cycles of combined immunotherapy and targeted maintenance therapy (Cadonilimab 250mg on days 1 and 8; Anlotinib 12mg daily from days 1 to 14, repeated every 3 weeks) initiated on May 30, 2023 and June 20, 2023. Following the detection of elevated transaminase levels (ALT 63 U/L, AST 89 U/L on July 10, 2023), the anlotinib dosage was reduced to 8mg. The patient subsequently received two cycles of maintenance therapy with immunotherapy and targeted agents on July 11 and August 7, 2023. Post-treatment surveillance revealed recurrent hepatic dysfunction, necessitating discontinuation of immunotherapy due to suspected immune-mediated hepatitis. The patient has been evaluated at three-month intervals through a standardized follow-up protocol comprising complete blood count, biochemical profiling, tumor marker assays, CT imaging, as well as assessments of Minimal Residual Disease (MRD) and Circulating Tumor Cells (CTCs). To date, surveillance has not detected any evidence of disease recurrence or metastatic progression (**Figure 1D**). His postoperative convalescence is largely favorable, with stable oral intake and normal sleep patterns. Notably, a mild degree of weight loss has been observed, necessitating ongoing active follow-up.

Past Medical History: The patient has a long-standing history of hypertension spanning over a decade, managed effectively with daily amlodipine. He reports no other chronic illnesses, such as diabetes or coronary artery disease, and no prior history of infectious diseases including hepatitis or tuberculosis. There is no record of major trauma, surgical procedures, or allergies to food or medications. He received a blood transfusion in November 2022. His immunization status is complete and consistent with national guidelines. The patient also describes a 30-year history of alcohol use, with a peak daily consumption of roughly 250 ml.

Family History: His mother had a medical history of gastric cancer.

## Discussion and conclusion

The pathogenesis of SMARCA4 (BRG1)-deficient tumors is closely associated with the functional inactivation of the SWI/SNF chromatin remodeling complex (1). This article reports a case of a poorly differentiated-undifferentiated tumor primarily originating in the stomach, which harbored SMARCA4 deficiency. This represents the first case of SMARCA4-deficient gastric carcinoma documented at our center. The management and outcomes in this case contribute novel clinical insights toward developing effective therapeutic strategies for this class of tumors.

Owing to the absence of distinctive morphological features, diagnosing this tumor category requires an integrated approach that combines SMARCA4 immunohistochemistry with genetic sequencing for definitive classification (3). In our patient, a combined histological and molecular work-up revealed a poorly differentiated/undifferentiated carcinoma with rhabdoid features, which demonstrated loss of SMARCA4 protein expression and a corroborating SMARCA4 mutation. Currently, no consensus exists regarding the standard-of-care for SMARCA4-deficient tumors. Despite preliminary evidence supporting the potential of agents like EZH2 inhibitors (tazemetostat) and ATR inhibitors (9, 10), the overall prognosis for patients with SMARCA4-deficient tumors remains grave. The disease typically follows a rapidly progressive course, a clinical behavior reflected in the reported median overall survival, which is generally under one year (5, 6). Ifosfamide is a broad-spectrum antitumor agent. It is used in the treatment of various solid tumors, such as soft tissue sarcomas and osteosarcomas (11). It also has applications for certain rare neoplasms (11). Anlotinib is a multi-target tyrosine kinase inhibitor. It works by inhibiting several key signaling pathways, including vascular endothelial growth factor receptor (VEGFR), platelet-derived growth factor receptor (PDGFR), fibroblast growth factor receptor (FGFR), and c-Kit. This action suppresses tumor angiogenesis and tumor cell proliferation (12). Cadonilimab is a bispecific antibody targeting both PD-1 and CTLA-4. It was approved in China in June 2022 for the treatment of platinum-resistant recurrent or metastatic cervical cancer (13). Following MDT discussion, the postoperative treatment regimen was determined to be a combination of ifosfamide, liposomal doxorubicin, cadonilimab, and anlotinib.

Among the limited number of cases reported to date, Cheng et al. (14) described two patients treated with postoperative chemotherapy combined with a single PD-1 inhibitor (Sintilimab). No recurrence or metastasis was observed during their postoperative follow-up periods of 16 and 3 months, respectively. Following surgery, the patient in our case was managed with an intensive multimodal regimen combining chemotherapy, the PD-1/CTLA-4 bispecific antibody cadonilimab, and targeted therapy. Subsequent follow-up has shown no evidence of recurrence or metastasis. The postoperative relapse-free survival (RFS) has now reached 33 months, exceeding the duration reported in most existing literature for comparable cases. This favorable outcome offers a meaningful clinical reference for the management of patients with similar tumors.

The use of cadonilimab (a PD-1/CTLA-4 bispecific antibody) in this case is particularly illustrative of immunotherapy’s potential for SMARCA4-deficient gastric carcinoma (SDGC). It therefore argues for expanding investigative efforts to other immunotherapies—such as adoptive cell therapy, oncolytic virus therapy, and cytokines (15). Ultimately, unlocking these treatments depends on first mapping the distinct immune microenvironment of SDGC to inform precise therapeutic strategies.

The inherent limitation of this single-case observation underscores the need for broader, multi-center investigations to validate the reported therapeutic outcomes. Concurrently, elucidating the precise mechanistic role of SWI/SNF complex inactivation in gastric carcinogenesis is essential to guide the rational development of specific treatments.

## Data Availability

The datasets presented in this article are not readily available because of ethical and privacy restrictions. Requests to access the datasets should be directed to the corresponding author/s.
